# Supplementary material for: Stephania tetrandra and Its Active Compound Coclaurine Sensitize NSCLC Cells to Cisplatin through EFHD2 Inhibition
Source: Pharmaceuticals (Basel). 2024 Oct 11;17(10):1356. doi: 10.3390/ph17101356 (PMC11510146; doi:10.3390/ph17101356)

Stephania tetrandra and its effective compound coclaurine sensitize NSCLC cells to cisplatin through EFHD2 inhibition

Shu-Yu Hu, Tsai-Hui Lin, Chung-Yu Chen, Yu-Hao He, Wei-Chien Huang, Ching-Yun Hsieh, Ya-Huey Chen, Wei-Chao Chang.

Supplementary Figure S1 The original images of Western blotting

Fig. 1C

Cropped blots in main paper

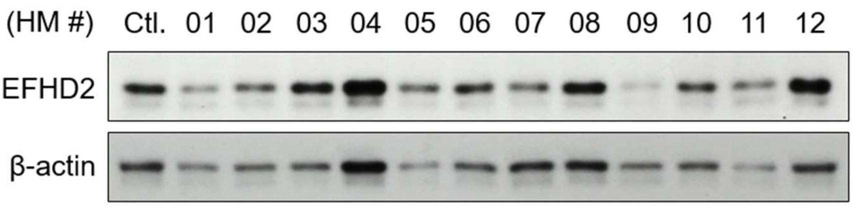

Original blots in supplementary information

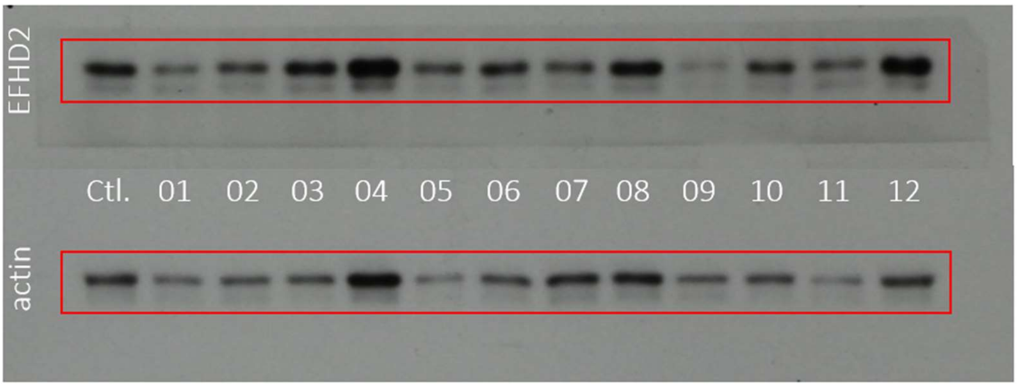

Fig. 2A

Cropped blots in main paper

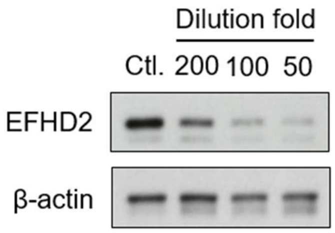

Original blots in supplementary information

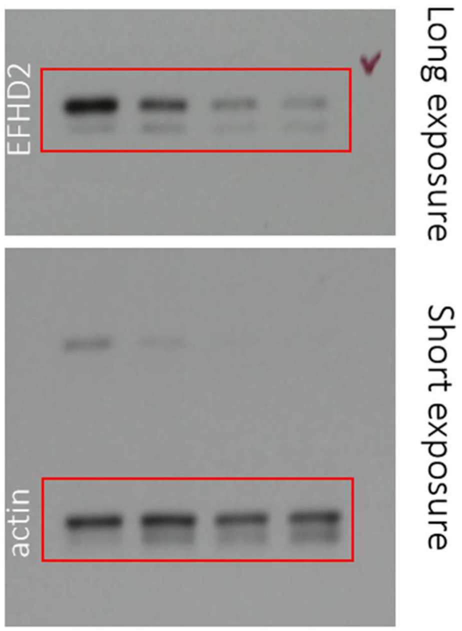

Fig. 2B

Cropped blots in main paper

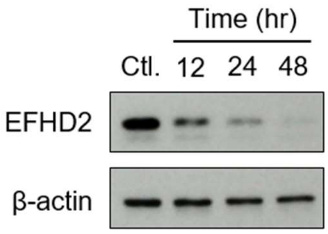

Original blots in supplementary information

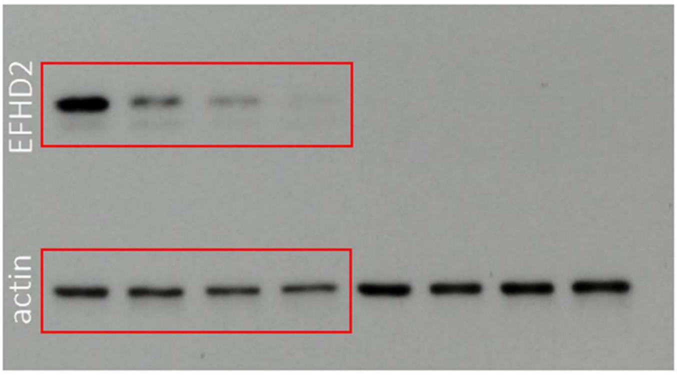

Fig. 3B

Cropped blots in main paper

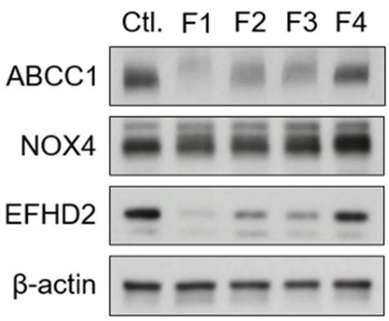

Original blots in supplementary information

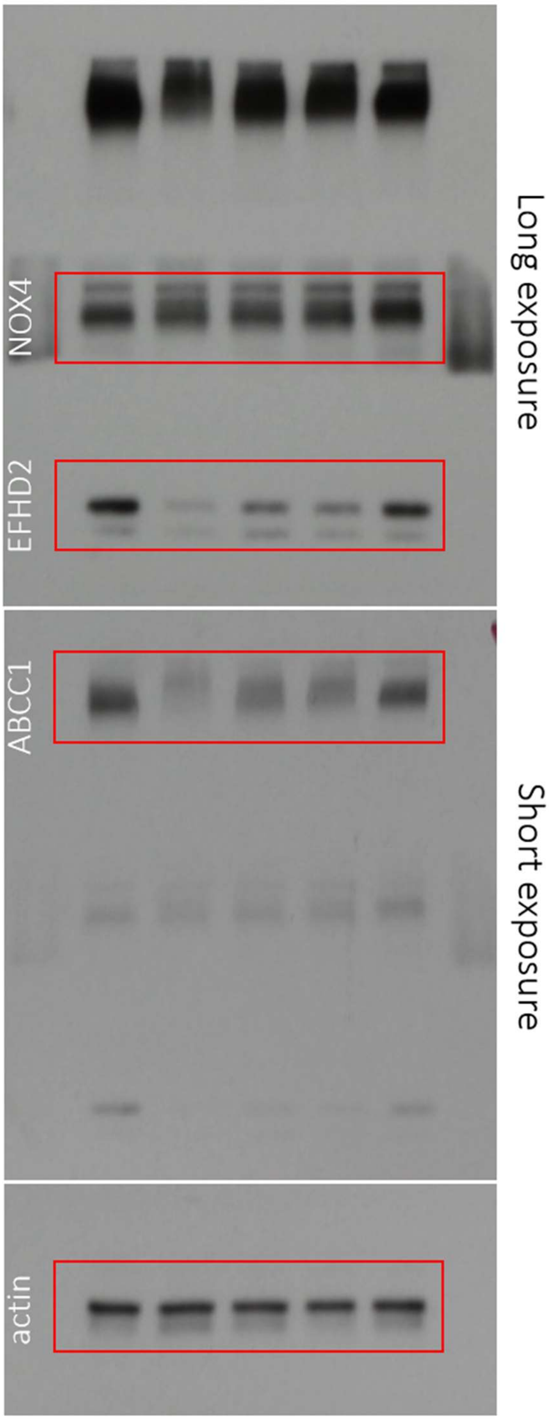

Fig. 3C

Cropped blots in main paper

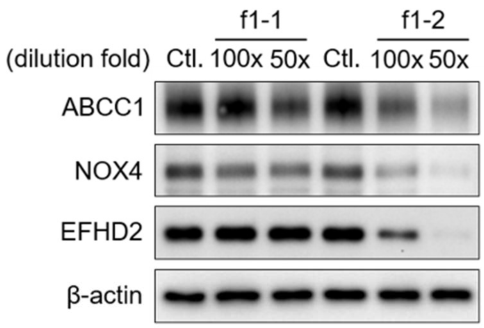

Original blots in supplementary information

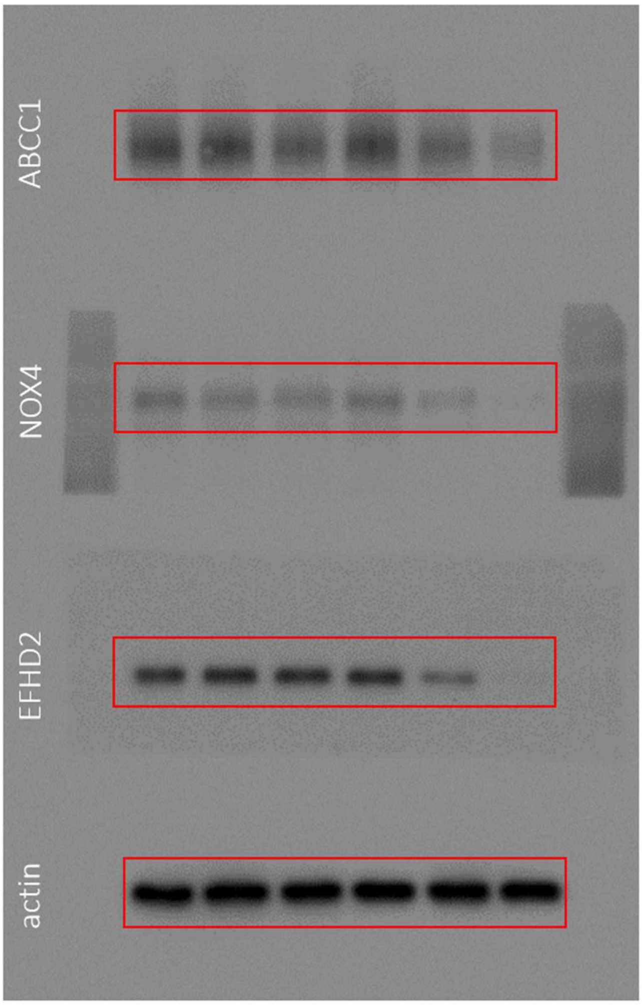

Fig. 4D

Cropped blots in main paper

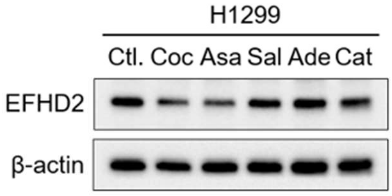

Original blots in supplementary information

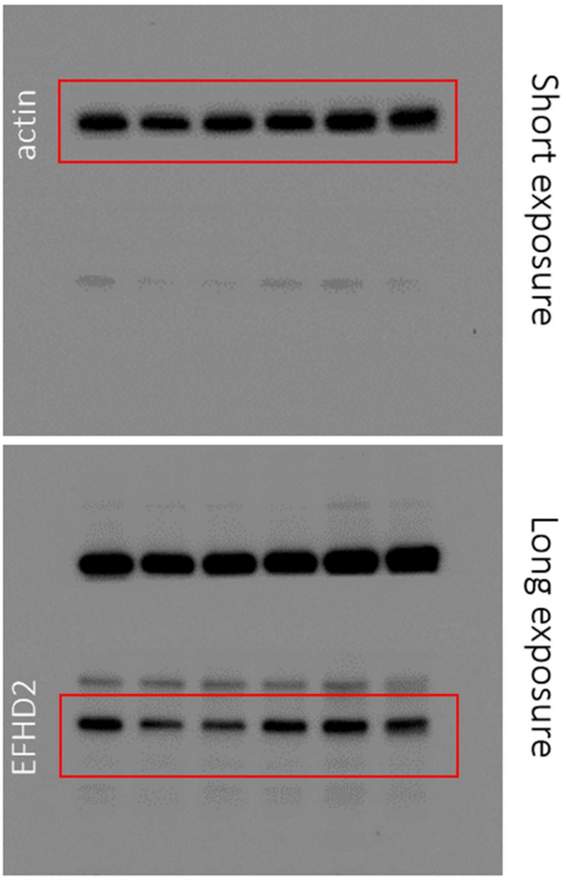

Fig. 5A

Cropped blots in main paper

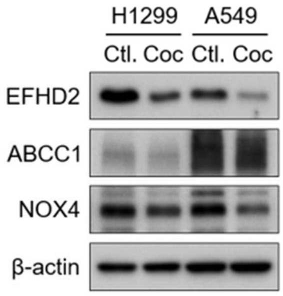

Original blots in supplementary information

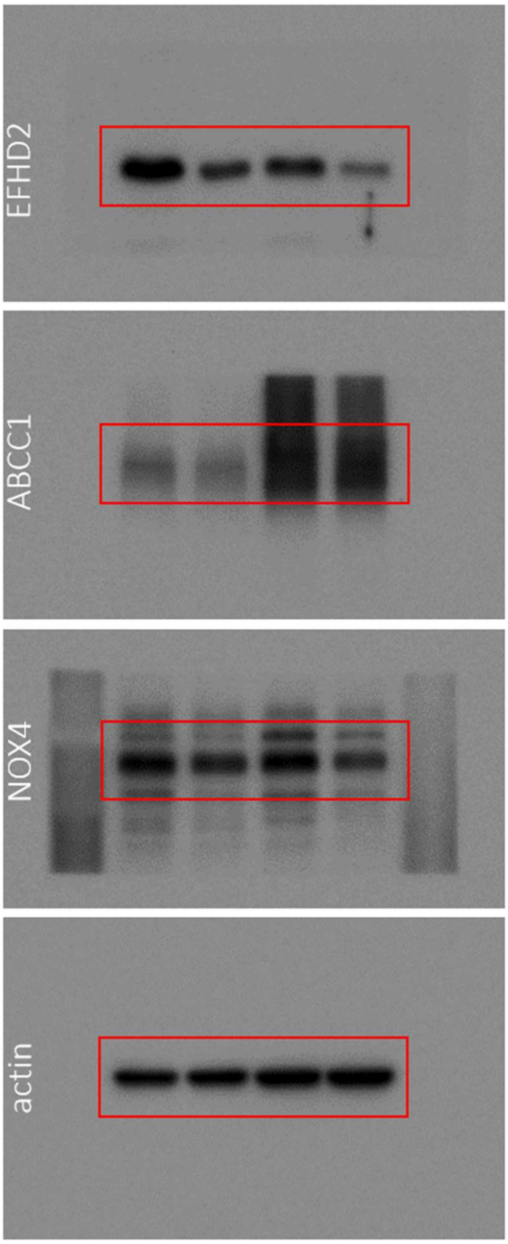

Fig. 5G

Cropped blots in main paper

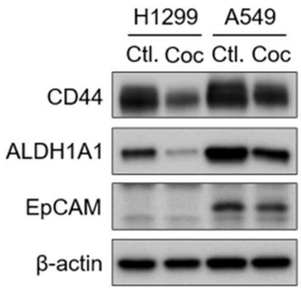

Original blots in supplementary information

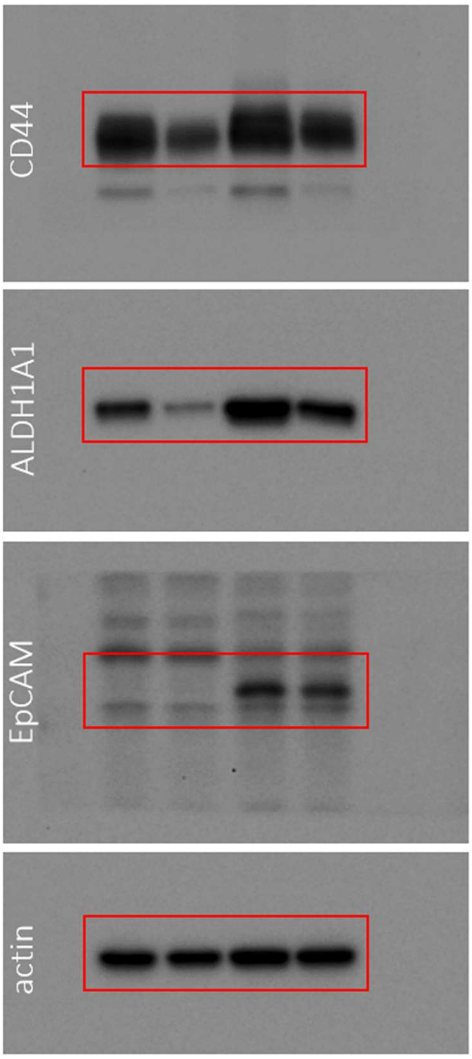

Fig. 6A

Cropped blots in main paper

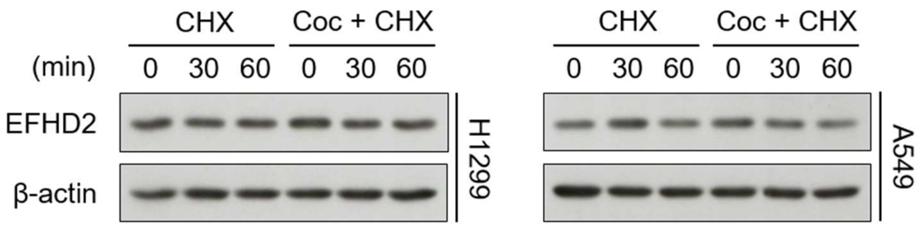

Original blots in supplementary information

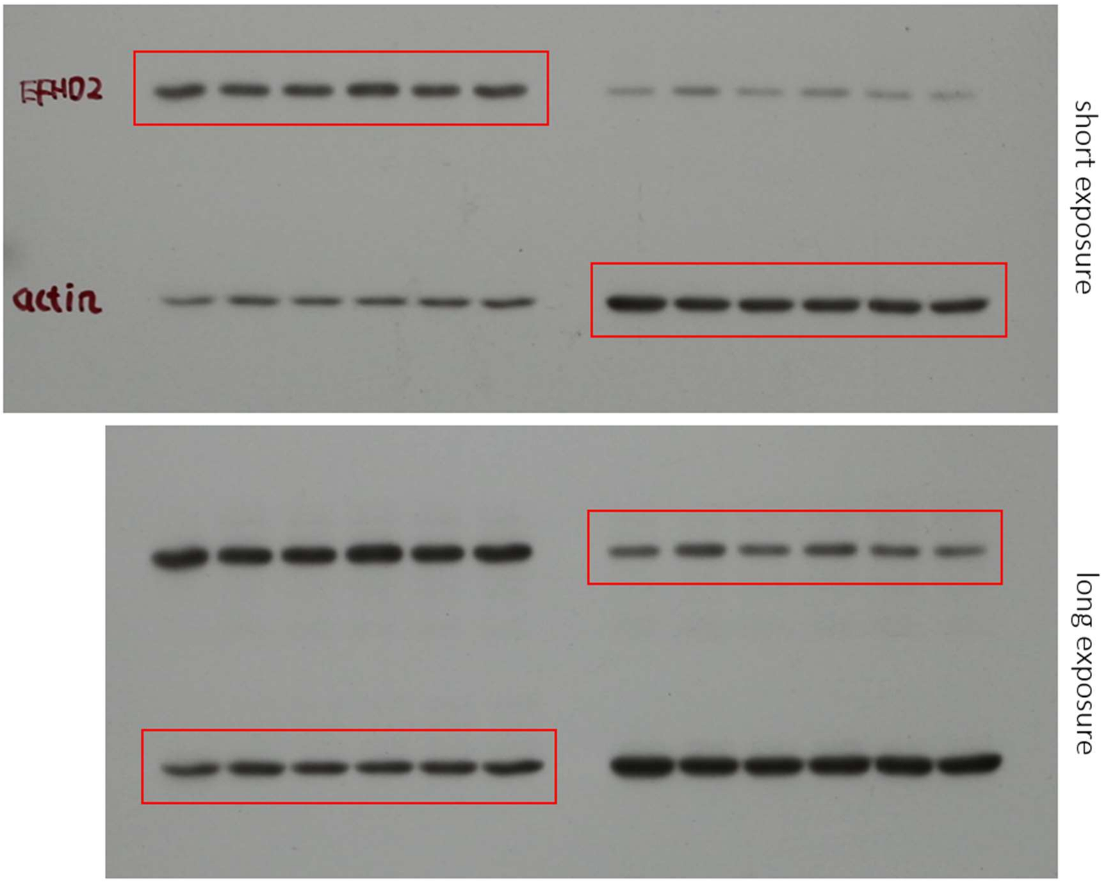

Fig. 6G

Cropped blots in main paper

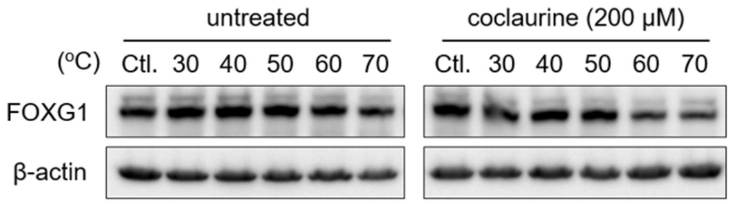

Original blots in supplementary information

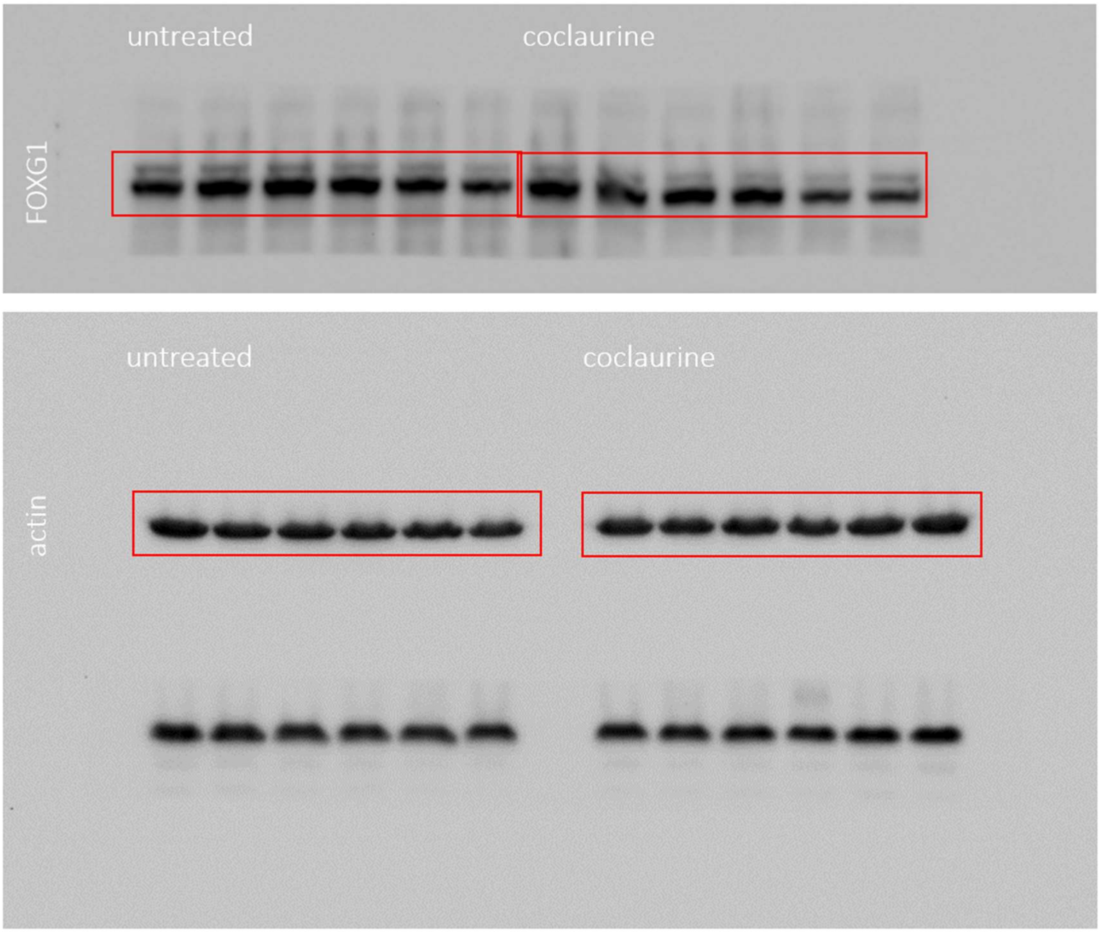

Fig. 6I

Cropped blots in main paper

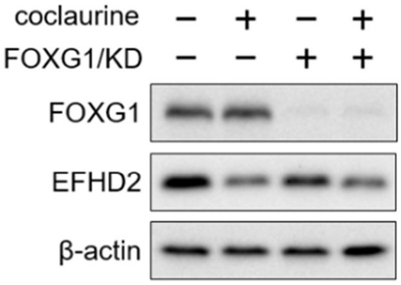

Original blots in supplementary information

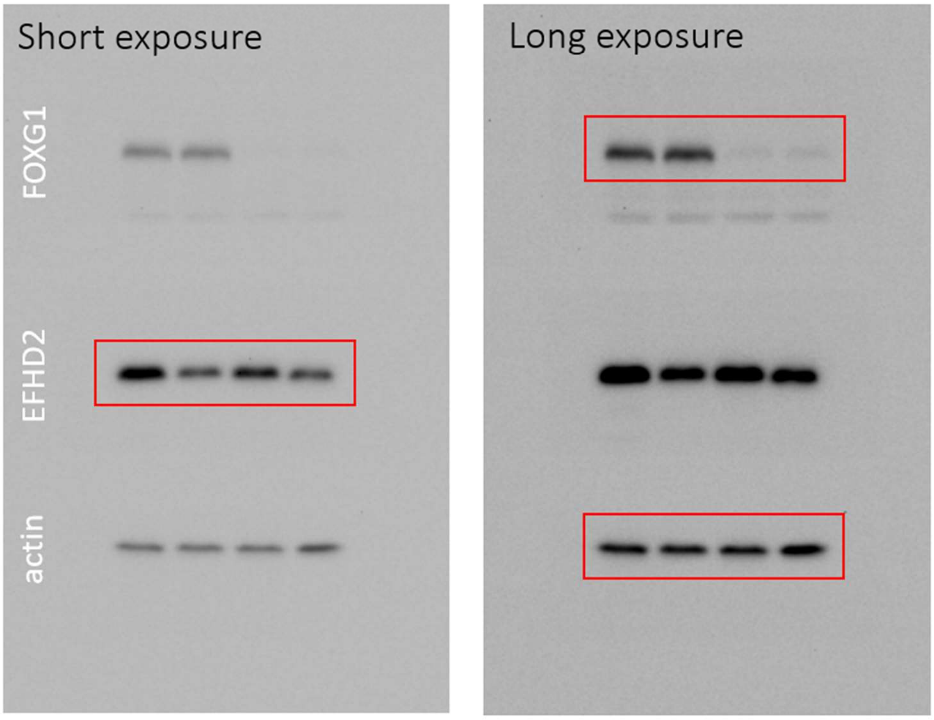

Supplement: Supplementary file 1 [file pharmaceuticals-17-01356-s001.zip › pharmaceuticals-3161957-supplementary/pharmaceuticals-3161957-supplementary.pdf]
